# Association between the stress hyperglycemia ratio and neovascular glaucoma in patients with proliferative diabetic retinopathy

**DOI:** 10.3389/fendo.2026.1945179

**Published:** 2026-09-28

**Authors:** Chenwei Zhao, Jiahao Xu, Hongmei Qian, Man Luo

**Affiliations:** Department of Ophthalmology, Shaoxing People’s Hospital, Shaoxing, Zhejiang, China

**Keywords:** acute-on-chronic glycemic discrepancy, diabetes complications, diabetic retinopathy, neovascular glaucoma, proliferative diabetic retinopathy, stress hyperglycemia, stress hyperglycemia ratio

## Abstract

**Background:**

Neovascular glaucoma (NVG) is a sight-threatening complication of proliferative diabetic retinopathy (PDR). The stress hyperglycemia ratio (SHR), which quantifies the discrepancy between acute admission glucose and chronic glycemic background, has been associated with adverse outcomes in diabetic populations, but its relationship with NVG remains unknown.

**Objective:**

To examine the association between the SHR and prevalent NVG among patients with PDR.

**Methods:**

In this retrospective cross-sectional study, patients with PDR presenting to Shaoxing People’s Hospital between 2023 and 2025 were categorized according to NVG status (49 with NVG; 215 without). The SHR was calculated as admission blood glucose (mmol/L) ÷ (1.59 × HbA1c − 2.59). Multivariable logistic regression estimated adjusted odds ratios (aORs), with the per-0.1-unit SHR increment as the primary effect measure; Firth penalized regression and a model excluding HbA1c were fitted as sensitivity analyses. Non-normally distributed variables were compared using the Mann–Whitney U test, with a Bonferroni-adjusted threshold of 0.0063 for the eight baseline comparisons. Discrimination was internally validated by bootstrap resampling.

**Results:**

Patients with NVG were younger (median 57.0 vs. 62.0 years; p < 0.001) and had higher SHR values (median 0.96 vs. 0.82; p < 0.001). The SHR was independently associated with prevalent NVG (aOR per 0.1-unit increment 1.31, 95% CI 1.12–1.53; p < 0.001); the Firth-penalized estimate was nearly identical (aOR 1.29, 95% CI 1.11–1.50). The events-per-variable ratio was 6.1; subgroup estimates were statistically unstable and are reported as exploratory only. The optimism-corrected C-statistic was 0.724.

**Conclusions:**

In this cross-sectional analysis, an elevated SHR was associated with a higher prevalence of NVG among patients with PDR. Because established NVG may itself increase admission glucose through acute stress, reverse causation cannot be excluded. These hypothesis-generating findings require confirmation in prospective, externally validated cohorts before any clinical application can be considered.

## Introduction

Diabetic retinopathy (DR) is a leading cause of vision loss among working-age adults, with proliferative diabetic retinopathy (PDR) representing its most advanced and sight-threatening stage (1). The complications of PDR extend beyond visual impairment: neovascular glaucoma (NVG) emerges as a particularly devastating sequela, characterized by aberrant neovascularization of the iris and iridocorneal angle that results in elevated intraocular pressure and progressive optic nerve damage (2, 3). Despite advances in the management of diabetic eye disease, NVG remains a major cause of irreversible blindness in this population.

The stress hyperglycemia ratio (SHR) is an emerging biomarker that captures the discrepancy between acute hyperglycemia at presentation and the underlying chronic glycemic background, and it has been associated with adverse outcomes in patients with diabetes (4). Unlike isolated measurements of blood glucose or glycated hemoglobin (HbA1c), the SHR indexes admission glucose against the estimated average glucose derived from HbA1c, thereby quantifying relative (stress) hyperglycemia — that is, an acute-on-chronic glycemic discrepancy (5). Importantly, the SHR is derived from a single glucose measurement and therefore does not capture within-patient glycemic variability, which requires serial glucose measurements or continuous glucose monitoring. Prior studies have reported associations between the SHR and cardiovascular mortality, renal outcomes, and outcomes of critical illness in diabetic cohorts (6, 7). However, the association between the SHR and NVG among patients with PDR remains largely unexplored.

Identifying factors associated with NVG secondary to PDR is essential for understanding its clinical correlates and for generating hypotheses regarding surveillance strategies. Although conventional risk factors — including suboptimal long-term glycemic control, prolonged diabetes duration, and systemic hypertension — are well established, the contribution of acute-on-chronic glycemic discrepancy remains poorly characterized (8). Notably, diabetic retinopathy and chronic kidney disease share common microvascular pathophysiological mechanisms and risk profiles, with inadequate glycemic management representing a central element (9). Given that the SHR has been associated with adverse renal outcomes in diabetic patients (10), it is biologically plausible that this biomarker may also be related to advanced diabetic retinal disease and its sight-threatening complications.

We hypothesized that higher SHR values would be independently associated with the presence of NVG in patients with PDR. The present cross-sectional study was therefore designed to examine the association between the SHR and prevalent NVG and to explore potential demographic and clinical modifiers of this association in an explicitly hypothesis-generating framework.

## Methods

### Study design and population

This retrospective cross-sectional study investigated the association between the stress hyperglycemia ratio (SHR) and neovascular glaucoma (NVG) in patients with proliferative diabetic retinopathy (PDR). The cohort was assembled through a retrospective review of the medical records of consecutive patients with PDR who presented to Shaoxing People’s Hospital between January 2023 and December 2025. Patients were eligible if PDR was confirmed according to the criteria below and the data required to compute the SHR were available. Records of 266 patients with PDR (51 with NVG and 215 without NVG) were identified. Two patients — both in the NVG group — were excluded from the analysis because one key covariate was missing (diabetes duration in one patient and serum creatinine in the other), yielding a final complete-case analytical cohort of 264 patients: 49 with NVG (cases) and 215 without NVG (controls). The patient-selection process is summarized in **Figure 1**.

**Figure 1:**
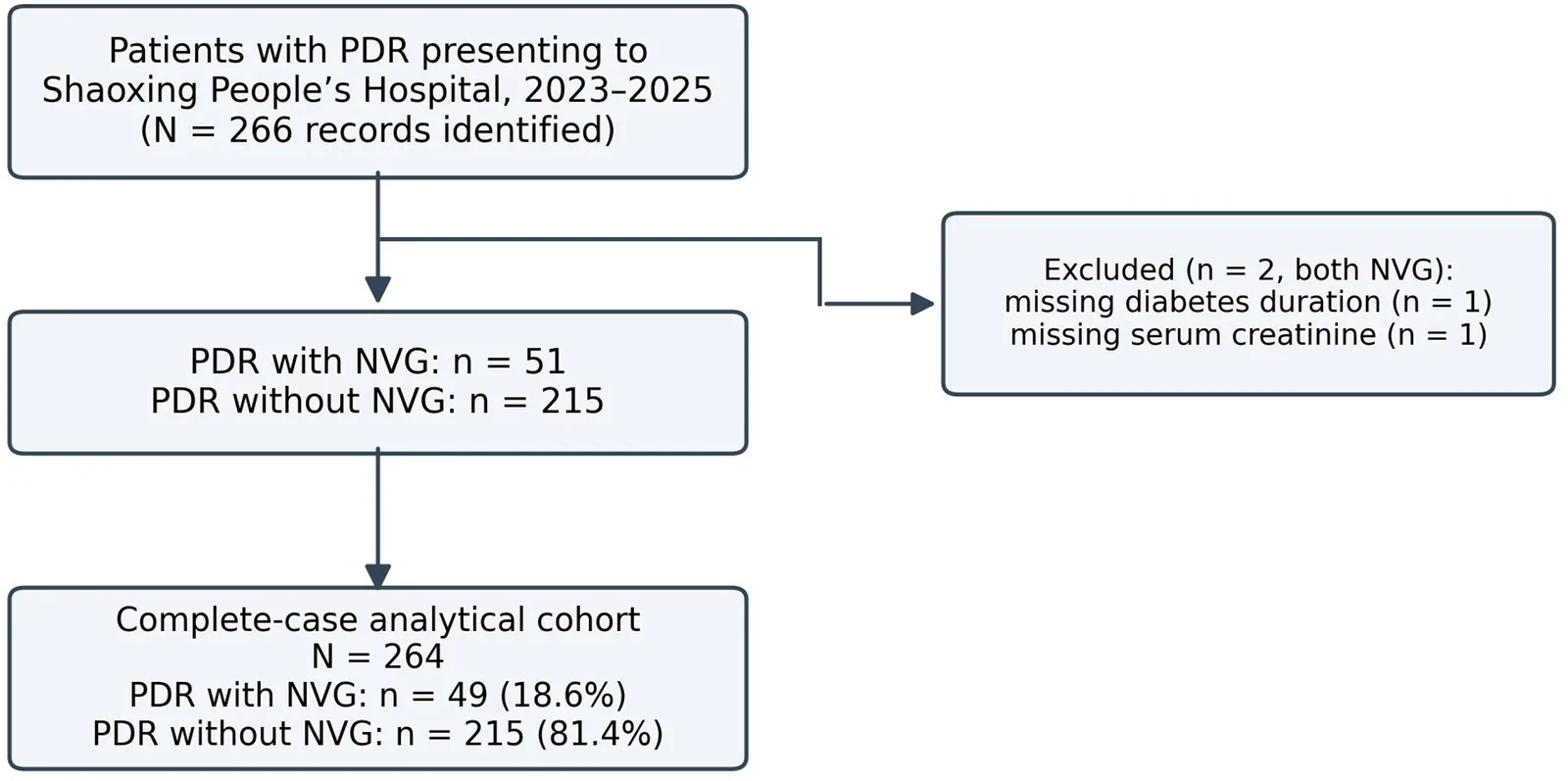
Patient-selection flow diagram. Patients were consecutively included if they met all eligibility criteria. Records of 266 patients with PDR were identified through retrospective review (51 with NVG, 215 without NVG). Two patients — both with NVG — were excluded because one key covariate was missing (diabetes duration, n = 1; serum creatinine, n = 1). The final complete-case analytical cohort comprised 264 patients (49 with NVG, 215 without NVG).

The analytical unit was the patient (one observation per patient). For patients with bilateral PDR, the eye with the more advanced stage of disease was used for classification according to the following hierarchy: (1) presence of NVG (iris neovascularization and/or angle neovascularization with elevated intraocular pressure); (2) presence of neovascularization of the optic disc (NVD); (3) presence of neovascularization elsewhere (NVE); (4) presence of vitreous hemorrhage or preretinal hemorrhage. The presence of NVG in either eye classified the patient as a case (PDR with NVG). Patients with bilateral NVG were classified identically to those with unilateral NVG. No patient contributed more than one observation to the analysis. Clinical and laboratory data (e.g., blood glucose, HbA1c, serum creatinine) were patient-level measurements and were not eye-specific.

The study protocol was approved by the Institutional Review Board of Shaoxing People’s Hospital (approval number 2025 Scientific research project 004-Y-01). Given the retrospective design based on de-identified routinely collected clinical data, the requirement for individual informed consent was waived by the committee on the basis of minimal risk and the impracticability of obtaining consent for retrospective record review, in accordance with institutional policy and the Declaration of Helsinki. All data were de-identified prior to analysis by removal of direct patient identifiers (name, medical record number, date of birth, contact information) and replacement with non-reversible study-specific numeric codes.

### Data collection

The following variables were extracted from the medical records: demographic characteristics (age and sex), diabetes duration, glycated hemoglobin (HbA1c), admission blood glucose, systolic blood pressure (SBP), diastolic blood pressure (DBP), and serum creatinine. Admission blood glucose was defined as the first venous plasma glucose measurement obtained within 24 hours of inpatient admission to the ophthalmology department. This was a random (non-fasting) sample, collected at the time of presentation with ocular symptoms prompting hospital admission, and before the initiation of any ocular treatment (e.g., intravitreal anti-VEGF injection, laser photocoagulation) or systemic therapy (e.g., intravenous mannitol, oral or intravenous hypoglycemic agents). For patients presenting with acute NVG-related symptoms (e.g., ocular pain, vision loss, headache), the admission glucose was obtained before NVG was formally diagnosed by slit-lamp examination and gonioscopy, although clinical suspicion of NVG may have prompted the encounter. For patients with previously diagnosed NVG presenting for readmission due to disease progression or complication management, the glucose was obtained at the time of that admission, prior to any treatment modification. In all cases, the admission glucose reflected the glycemic status at presentation rather than a fasting baseline. All glucose values were expressed in mmol/L and HbA1c in %. The SHR was calculated using the formula proposed by Roberts et al. (5): admission glucose (mmol/L) ÷ (1.59 × HbA1c − 2.59), in which the denominator yields the estimated average glucose in mmol/L according to the relationship established by Nathan et al. (11); the numerator and denominator were therefore expressed in identical units before division. In the present cohort, HbA1c ranged from 4.3% to 14.9%, the denominator remained positive for all observations (minimum value 4.25), and no invalid values required exclusion.

Because NVG is itself a severe acute stressor that can increase admission glucose, the temporal relationship between glucose measurement and NVG status is central to interpretation. Glucose and HbA1c values used here were obtained at the admission during which NVG status was ascertained before the initiation of any therapy that could acutely alter blood glucose, including: (1) ocular treatments (e.g., intravitreal anti-VEGF injection, laser photocoagulation, cyclophotocoagulation); (2) osmotic therapy (e.g., intravenous mannitol); (3) corticosteroid administration (e.g., systemic, periocular, or intravitreal); and (4) initiation or escalation of hypoglycemic agents. The potential for reverse causation is addressed explicitly in the Discussion and Limitations.

### Outcome definition

The diagnosis of proliferative diabetic retinopathy (PDR) was established according to the standardized clinical grading criteria of the Early Treatment Diabetic Retinopathy Study (ETDRS). PDR was defined by the presence of neovascularization elsewhere (NVE), neovascularization of the optic disc (NVD), vitreous hemorrhage, or preretinal hemorrhage (12). Neovascular glaucoma (NVG) was diagnosed based on the presence of iris neovascularization (rubeosis iridis) and/or anterior chamber angle neovascularization, accompanied by elevated intraocular pressure (> 21 mmHg), in the context of proliferative diabetic retinopathy.

### Statistical analysis

The distribution of each continuous variable was assessed using the Shapiro–Wilk test andvisual inspection of histograms (**Supplementary Figure 1**). Because most continuous variables — including serum creatinine — deviated from normality, continuous variables are summarized as median (interquartile range [IQR]) and compared using the Mann–Whitney U test. Categorical variables are expressed as frequencies and percentages and compared using chi-square (χ²) tests. For the baseline comparison of eight characteristics, a prespecified Bonferroni correction was applied, yielding an adjusted two-sided significance threshold of 0.0063 (0.05/8); the same correction family was applied consistently throughout the descriptive analyses.

Logistic regression was used to estimate the association between the SHR and NVG, with results reported as odds ratios (ORs) and 95% confidence intervals (CIs). Because the observed SHR range in this cohort was 0.10–1.72, the per-0.1-unit increment in SHR was prespecified as the primary, clinically interpretable effect measure; per-1-unit estimates are additionally reported for completeness but represent extrapolation beyond the observed data range. Three modeling approaches were used: (1) an unadjusted model with the SHR as the sole predictor; (2) a multivariable model additionally incorporating age, sex, diabetes duration, HbA1c, SBP, DBP, and serum creatinine; and (3) subgroup analyses stratified by sex, age (≤60 vs. >60 years), and diabetes duration (≤10 vs. >10 years), with multiplicative interaction terms. The subgroup cutoffs for age (≤60 vs. >60 years) and diabetes duration (≤10 vs. >10 years) were chosen *a priori*, before any data analysis, as conventional clinical thresholds in diabetic complication research. The 10-year diabetes duration cutoff is widely used to distinguish early-stage from long-standing disease and corresponds exactly to the cohort median duration (10.0 years); the 60-year age cutoff approximates the cohort median age (62.0 years) and is consistent with age strata commonly employed in ophthalmic and metabolic complication studies.

Several analyses were performed to address statistical stability. The events-per-variable (EPV) ratio was computed (49 events/8 covariates; EPV = 6.1). Because the number of NVG events was limited relative to the number of covariates, Firth penalized logistic regression (Jeffreys-prior penalized likelihood) was fitted for the main model and all subgroup models to reduce small-sample and sparse-data bias; estimates derived from small event counts, including all subgroup estimates, are explicitly labeled as unstable and exploratory. Variance inflation factors (VIFs) were calculated to assess multicollinearity, which is relevant because HbA1c contributes mathematically to the SHR denominator. To examine this mathematical coupling directly, three sensitivity models were fitted: (a) the full multivariable model excluding HbA1c; (b) a model adjusting for admission glucose instead of the SHR; and (c) a model adjusting for HbA1c instead of the SHR, to determine whether the SHR carries information beyond its individual components. Serum creatinine was rescaled per 50 μmol/L for clinical interpretability.

Model discrimination was quantified using the C-statistic (area under the receiver operating characteristic curve). Because discrimination was evaluated in the same dataset used for model fitting, internal validation was performed with 1,000 bootstrap resamples to obtain optimism-corrected C-statistics with 95% CIs, and model calibration was assessed using the Hosmer–Lemeshow test. These discrimination analyses are presented as exploratory rather than as evidence of validated clinical prediction. All analyses were performed using Python (version 3.12.14) with statsmodels (version 0.15.0) and scipy (version 1.18.1); Firth regression was implemented with a custom Jeffreys-prior penalized-likelihood routine included in the analysis script. The de-identified dataset and the complete analysis script are available at 10.5281/zenodo.22332660.

## Results

### Patient characteristics

Records of 266 patients with PDR were identified; after exclusion of two patients with one missing covariate each, the final analytical cohort comprised 264 patients, of whom 49 (18.6%) had PDR complicated by NVG and 215 (81.4%) had PDR without NVG (**Figure 1**). Baseline characteristics are summarized in **Table 1**; distributions of serum creatinine and the SHR are shown in **Supplementary Figure 1**.

**Table 1:** Baseline characteristics of the study population.

Characteristic	Total (N = 264)	PDR with NVG (n = 49)	PDR without NVG (n = 215)	p-value
Age, years	62.0 (53.0–70.0)	57.0 (48.0–63.0)	62.0 (55.0–70.5)	<0.001
Male, n (%)	153 (58.0)	32 (65.3)	121 (56.3)	0.320
Diabetes duration, years	10.0 (8.0–20.0)	10.0 (10.0–18.0)	10.0 (7.0–20.0)	0.498
HbA1c, %	8.3 (6.8–10.1)	8.2 (7.0–10.7)	8.3 (6.8–10.1)	0.931
SBP, mmHg	138.0 (126.0–149.2)	139.0 (126.0–155.0)	137.0 (127.0–148.5)	0.720
DBP, mmHg	80.0 (74.8–87.2)	82.0 (77.0–89.0)	80.0 (73.5–87.0)	0.163
Serum creatinine, μmol/L	64.8 (52.5–82.4)	78.1 (61.6–126.0)	63.3 (50.4–78.7)	<0.001
SHR	0.83 (0.71–0.98)	0.96 (0.78–1.10)	0.82 (0.70–0.95)	<0.001

Continuous variables are expressed as median (interquartile range) because each continuous variable deviated from normality in at least one group on the Shapiro–Wilk test (p < 0.05; distributions in **Supplementary Figure 1**) and were compared using the Mann–Whitney U test; the categorical variable was compared using the chi-square test. A prespecified Bonferroni correction across the eight baseline comparisons yielded an adjusted significance threshold of 0.0063.

Patients with NVG were significantly younger than those without NVG (median 57.0 vs. 62.0 years; p < 0.001), had higher SHR values (median 0.96 vs. 0.82; p < 0.001), and had higher serum creatinine (median 78.1 vs. 63.3 μmol/L; p < 0.001). All three differences remained significant after Bonferroni correction (adjusted threshold 0.0063). No significant intergroup differences were observed in sex distribution, diabetes duration, HbA1c, SBP, or DBP.

### Association between the SHR and NVG

After adjustment for age, sex, diabetes duration, HbA1c, SBP, DBP, and serum creatinine, each 0.1-unit increment in the SHR was associated with 31% higher odds of prevalent NVG (adjusted odds ratio [aOR] 1.31, 95% CI 1.12–1.53; p < 0.001; **Table 2**). The full set of adjusted estimates from the multivariable model is presented in **Table 3**. The adjusted per-1-unit estimate (aOR 14.91) exceeded the unadjusted estimate (OR 10.83); this is attributable to negative confounding by age, which was inversely associated with NVG and correlated with the SHR, such that adjustment for age and the other covariates strengthened the estimated association. The events-per-variable ratio was 6.1, below the conventional threshold of 10; the Firth-penalized estimate was nearly identical to the maximum-likelihood estimate (aOR 1.29, 95% CI 1.11–1.50; p < 0.001), indicating that the association is robust to sparse-data bias, although precision remains limited by the modest number of events.

**Table 2:** Association between the SHR and NVG: logistic regression results.

Model	OR per 0.1-unit SHR (95% CI)	p-value	C-statistic (95% CI)
Unadjusted	1.27 (1.10–1.46)	<0.001	0.657 (0.562–0.742)
Adjusted	1.31 (1.12–1.53)	<0.001	0.769 (0.721–0.851)
Adjusted, Firth penalized	1.29 (1.11–1.50)	<0.001	—
Adjusted, excluding HbA1c	1.28 (1.10–1.48)	0.001	—

The primary effect measure is the per-0.1-unit increment in SHR. Optimism-corrected C-statistics from 1,000 bootstrap resamples were 0.659 (unadjusted) and 0.724 (adjusted); Hosmer–Lemeshow calibration p-values were 0.905 and 0.092, respectively. Per-1-unit estimates (unadjusted OR 10.83, 95% CI 2.69–43.58; adjusted OR 14.91, 95% CI 3.20–69.53) are reported for completeness but extrapolate beyond the observed SHR range (0.10–1.72) and should be interpreted with caution.

**Table 3:** Full multivariable logistic regression results.

Variable	Adjusted OR	95% CI	p-value
SHR (per 0.1 unit)	1.31	1.12–1.53	<0.001
Age (per year)	0.96	0.92–0.99	0.022
Male sex	1.11	0.54–2.29	0.771
Diabetes duration (per year)	1.02	0.97–1.07	0.392
HbA1c (per %)	1.11	0.95–1.30	0.205
SBP (per mmHg)	1.00	0.98–1.02	0.968
DBP (per mmHg)	1.01	0.96–1.05	0.713
Serum creatinine (per 50 μmol/L)	1.18	1.05–1.32	0.007

Serum creatinine was rescaled per 50 μmol/L for clinical interpretability.

Variance inflation factors did not indicate problematic multicollinearity (all VIFs ≤ 2.14, including SHR at 1.13 and HbA1c at 1.15). In sensitivity analyses addressing the mathematical coupling between the SHR and HbA1c, the SHR remained significantly associated with NVG when HbA1c was removed from the adjustment set (aOR per 0.1 unit 1.28, 95% CI 1.10–1.48; p = 0.001). In component models (**Table 4**), admission glucose replacing the SHR was positively associated with NVG (aOR per 1 mmol/L 1.24, 95% CI 1.09–1.41; p = 0.001), whereas HbA1c replacing the SHR was not (aOR per 1% 1.02, 95% CI 0.88–1.19; p = 0.750), suggesting that the SHR association is carried predominantly by the acute admission-glucose component rather than by chronic glycemia. The fully adjusted model showed a C-statistic of 0.769 (optimism-corrected 0.724, bootstrap 95% CI 0.721–0.851); the Hosmer–Lemeshow test did not indicate lack of fit (p = 0.092). These discrimination analyses are exploratory and do not constitute validated clinical prediction.

**Table 4:** Sensitivity analyses addressing the mathematical coupling between the SHR and HbA1c.

Model	Effect estimate (95% CI)	p-value
Full adjusted model (SHR per 0.1 unit, with HbA1c)	1.31 (1.12–1.53)	<0.001
Adjusted model excluding HbA1c (SHR per 0.1 unit)	1.28 (1.10–1.48)	0.001
Admission glucose replacing SHR (per 1 mmol/L)	1.24 (1.09–1.41)	0.001
HbA1c replacing SHR (per 1%)	1.02 (0.88–1.19)	0.750

All models adjust for age, sex, diabetes duration, SBP, DBP, and serum creatinine; the first model additionally adjusts for HbA1c. Variance inflation factors in the full model were all ≤ 2.14.

Advancing age was inversely associated with prevalent NVG (aOR per year 0.96, 95% CI 0.92–0.99; p = 0.022), consistent with the younger median age observed among NVG patients in this cohort. Serum creatinine was positively associated with NVG (aOR per 50 μmol/L 1.18, 95% CI 1.05–1.32; p = 0.007). HbA1c was not significantly associated with NVG in the fully adjusted model (aOR 1.11, 95% CI 0.95–1.30; p = 0.205). This null finding should be interpreted cautiously: because HbA1c contributes mathematically to the SHR denominator, its simultaneous inclusion as a covariate may distort both estimates, and a non-significant HbA1c coefficient does not by itself establish that acute-on-chronic glycemic discrepancy carries prognostic information beyond chronic glycemic control. The sensitivity analyses excluding HbA1c and examining the SHR components separately (**Table 4**) provide the more appropriate framework for this question.

### Subgroup analyses

Subgroup analyses were conducted as exploratory, hypothesis-generating analyses (**Table 5**).

**Table 5:** Subgroup analyses of the association between the SHR and NVG.

Subgroup	n	NVG events	SHR (mean ± SD)	Unadjusted OR (95% CI)	p-value	Adjusted OR (95% CI)	p-value	Interaction p
Overall	264	49	0.85 ± 0.23	1.27 (1.10–1.46)	<0.001	1.31 (1.12–1.53)	<0.001	—
Sex								0.432
Female	111	17	0.86 ± 0.24	1.35 (1.08–1.68)	0.008	1.24 (0.97–1.59)	0.079	
Male	153	32	0.84 ± 0.22	1.23 (1.02–1.47)	0.027	1.28 (1.05–1.57)	0.015	
Age								0.869
≤60 years	116	31	0.86 ± 0.23	1.28 (1.06–1.56)	0.012	1.35 (1.08–1.69)	0.007	
>60 years	148	18	0.83 ± 0.23	1.24 (1.01–1.53)	0.041	1.27 (1.01–1.59)	0.038	
Diabetes duration								0.081
≤10 years	151	26	0.85 ± 0.21	1.50 (1.19–1.90)	<0.001	1.59 (1.21–2.08)	<0.001	
>10 years	113	23	0.84 ± 0.26	1.13 (0.95–1.35)	0.169	1.18 (0.97–1.44)	0.096	

All ORs are expressed per 0.1-unit increment in SHR. Adjusted estimates were obtained by Firth penalized logistic regression; in the female stratum the maximum-likelihood model failed to converge owing to quasi-complete separation, which itself illustrates the sparse-data instability of these analyses. Stratum counts sum exactly to the analytical cohort of 264 patients and 49 NVG events. Interaction p-values derive from fully adjusted models including the SHR × modifier product term.

The association between the SHR and NVG was numerically larger in some strata — for example, among patients with diabetes duration ≤10 years (adjusted OR 1.59 per 0.1 unit) than >10 years (adjusted OR 1.18 per 0.1 unit) — but stratum-specific estimates were derived from as few as 17 events and remain imprecise. None of the interaction tests reached statistical significance (sex, p = 0.432; age, p = 0.869; diabetes duration, p = 0.081). Accordingly, these subgroup results are presented strictly as exploratory, and no conclusions regarding effect modification are drawn.

## Discussion

In this retrospective cross-sectional study of patients with PDR, a higher SHR was independently associated with the presence of NVG after adjustment for demographic and metabolic covariates. Because the design compares patients with and without established NVG, the findings demonstrate an association with prevalent NVG; they do not establish prediction of incident NVG, and causal language is avoided throughout. This distinction is particularly important because NVG itself — through pain, inflammation, and the physiological stress of hospitalization — can increase admission glucose and thereby the SHR, making reverse causation a plausible alternative explanation for the observed association.

These findings are consistent with prior evidence linking suboptimal glycemic control to diabetic complications, including NVG (13). As an integrative metric of acute-on-chronic glycemic discrepancy, the SHR offers a complementary perspective to isolated glucose or HbA1c measurements (6, 7). It should be emphasized that the SHR is not a measure of glycemic variability: it is derived from a single admission glucose value normalized to HbA1c-estimated average glucose, whereas true glycemic variability requires serial measurements or continuous glucose monitoring. Our findings therefore should not be interpreted as evidence regarding glucose fluctuation, and mechanistic arguments derived from the glycemic-variability literature can be applied to the present results only indirectly and with caution (14, 15).

Unlike previous studies focusing primarily on average glycemic indices, the present study specifically examined the SHR in relation to NVG. Simó-Servat et al. (16) showed that intensive glucose control reduces the risk of diabetic retinopathy but did not assess relative hyperglycemia. Our results raise the hypothesis that acute-on-chronic glycemic discrepancy may be relevant to advanced diabetic eye disease; whether it carries information beyond chronic glycemia cannot be firmly established here, particularly given the mathematical coupling between the SHR and HbA1c. The component analyses suggested that the association was carried predominantly by the admission-glucose component: admission glucose remained associated with NVG when modeled in place of the SHR, whereas HbA1c did not.

The non-significant association between HbA1c and NVG in the fully adjusted model warrants cautious interpretation. Because HbA1c is a component of the SHR denominator, entering both into the same model can distort each estimate and artificially attenuate the HbA1c coefficient; the null HbA1c finding therefore does not demonstrate that chronic glycemic control is unimportant, nor does it establish the superiority or independence of the SHR. Similarly, the non-significant association observed for SBP should not be over-interpreted, given the limited power and cross-sectional design.

The numerically stronger association among patients with shorter diabetes duration — if confirmed — would add to growing evidence of heterogeneity in diabetic complications (17, 18). However, the interaction test did not reach statistical significance (p = 0.081) and the stratum-specific estimates were derived from few events; we therefore present this pattern solely as a hypothesis for validation in larger, adequately powered prospective cohorts rather than as evidence of effect modification (19).

The following mechanistic considerations are presented explicitly as hypotheses for future investigation; no mechanistic variables were measured in the present study. Experimental literature on glucose fluctuation (not directly on the SHR) suggests that acute glycemic stress can potentiate oxidative stress and inflammatory cascades implicated in diabetic complications (20), including mitochondrial superoxide generation (21), NLRP3 inflammasome activation with caspase-1–dependent cytokine release (e.g., IL-1β, IL-18) (22), and endothelial nitric oxide synthase impairment with upregulation of adhesion molecules (e.g., ICAM-1, VCAM-1) (23). Activation of the polyol pathway and accumulation of advanced glycation end products have likewise been implicated in retinal neovascularization and vascular dysfunction (24, 25). Whether these pathways mediate any relationship between the SHR and NVG is unknown and requires experimental and prospective clinical study.

## Limitations

This study has several important limitations. First and most importantly, the cross-sectional design precludes establishing temporality: glucose and HbA1c were measured at the admission during which NVG status was ascertained, and established NVG — through ocular pain, inflammation, and the stress of hospitalization — may itself elevate admission glucose and the SHR. The observed association may therefore reflect reverse causation, and the SHR cannot be interpreted as a causal or predictive factor on the basis of these data. Second, unmeasured ophthalmologic and clinical confounding is substantial: we lacked data on PDR severity and duration, prior panretinal photocoagulation, intravitreal anti-VEGF therapy, prior vitrectomy or other ocular surgery, retinal ischemic burden, baseline intraocular pressure, diabetes type and treatment, and the presence of other major diabetic complications including nephropathy; each of these may influence both NVG status and metabolic variables. Third, the number of NVG events (n = 49) was modest relative to the eight model covariates (EPV = 6.1), limiting precision — most evident in the subgroup analyses, where one stratum model failed to converge under maximum likelihood — although the near-identical Firth-penalized estimates mitigate concerns about bias in the main model. Fourth, the analysis relied on a single determination of the SHR rather than longitudinal trajectories. Fifth, the single-center, retrospective sampling frame may limit generalizability and may introduce selection bias. Finally, discrimination was assessed in the derivation dataset; although bootstrap internal validation was performed, external validation is absent.

Because time-to-event data on NVG onset were unavailable, Cox proportional hazards modeling was not feasible. Prospective cohort studies with longitudinal follow-up, systematic ascertainment of incident NVG, serial glucose measurements, and capture of ophthalmologic treatment variables are needed to evaluate any temporal relationship between the SHR and incident NVG.

## Clinical implications

The present findings are hypothesis-generating and should not yet inform clinical practice. If confirmed prospectively with external validation, the SHR — which is readily calculated from routinely available admission glucose and HbA1c — could potentially be investigated as one component of risk-stratification research in patients with PDR. At present, no change to surveillance or treatment protocols is warranted on the basis of this study.

## Conclusion

In this cross-sectional analysis, an elevated stress hyperglycemia ratio was independently associated with a higher prevalence of neovascular glaucoma among patients with proliferative diabetic retinopathy, an association that was robust to Firth penalization and to exclusion of HbA1c from the adjustment set. Given the cross-sectional design, the possibility of reverse causation, residual confounding by unmeasured ophthalmologic factors, and the limited number of events, this association should be regarded as hypothesis-generating. Prospective, externally validated longitudinal studies are required before the SHR can be considered for any role in risk stratification for NVG.

## Data Availability

The raw data supporting the conclusions of this article will be made available by the authors, without undue reservation.

## Supplementary material

Supplementary Figure 1 Distributions of serum creatinine **(A)** and the stress hyperglycemia ratio **(B)** by NVG status. Serum creatinine was markedly right-skewed in both groups, supporting the use of median (IQR) and the Mann–Whitney U test.
